# The Anti-Photoaging Activity of Peptides from *Pinctada martensii* Meat

**DOI:** 10.3390/md20120770

**Published:** 2022-12-08

**Authors:** Mengfen Wei, Huamai Qiu, Jie Zhou, Chenghao Yang, Yifan Chen, Lijun You

**Affiliations:** 1 School of Food Science and Engineering, South China University of Technology, Guangzhou 510640, China; 2 Guangzhou Institute of Modern Industrial Technology, Guangzhou 511458, China; 3 Ira A. Fulton Schools of Engineering, Arizona State University, Tempe, AZ 85281, USA

**Keywords:** *Pinctada martensii*, peptide, photoaging, HaCaT, molecular docking

## Abstract

Long-term exposure to ultraviolet-B (UVB) can cause photoaging. Peptides from *Pinctada martensii* meat have been shown to have anti-photoaging activities, but their mechanism of action is rarely studied. In this study, *Pinctada martensii* meat hydrolysates (PME) were prepared by digestive enzymes and then separated by ultrafiltration and Sephadex G-25 gel filtration chromatography to obtain a purified fraction (G2). The fraction G2 was identified by ultra-performance liquid chromatography tandem mass spectrometry (UHPLC-MS/MS), and peptide sequences were synthesized by solid-phase synthesis. The mechanism of anti-photoaging activities was investigated using a human immortalised epidermal (HaCaT) cell model. Results showed that peptides from *Pinctada martensii* meat increased UVB-induced cell viability and reduced the contents of interstitial collagenase (MMP-1) and matrix lysing enzyme (MMP-3) in HaCaT cells. Furthermore, the fraction of G2 significantly downregulated the expression of *p38*, *EKR*, *JNK*, *MMP-1*, and *MMP-3* in HaCaT cells. The peptide sequences Phe-His (FH), Ala-Leu (AL), Met-Tyr (MY), Ala-Gly-Phe (AGF), and Ile-Tyr-Pro (IYP) were identified and synthesized. Besides, FH reduced the contents of MMP-1 and MMP-3 in HaCaT cells, combining them effectively in molecular docking analysis. Thus, peptides from *Pinctada martensii* meat showed anti-photoaging activities and might have the potential to be used as an anti-photoaging agent in functional foods.

## 1. Introduction

Skin aging mainly includes intrinsic aging and extrinsic aging. Extrinsic aging can be caused by several extrinsic factors, such as air pollution, cigarette smoking, and ultraviolet (UV) irradiation [1], and UV irradiation leads to photoaging. UV irradiation mainly includes UVA (320–400 nm) and UVB (290–320 nm), which primarily cause damage to the dermis and epidermis, respectively [2]. Reactive oxygen species (ROS) can be induced by UV irradiation and then activate signaling pathways that result in the production of matrix metalloproteinases (MMPs), which are released from epidermal keratinocytes and dermal fibroblasts [3]. Collagen and elastin fibers, which are responsible for the structure and physiological functions of the skin, are degraded by MMPs in the extracellular matrix (ECM) when exposed to UV irradiation [4], leading to the formation of wrinkles in the skin. Therefore, exploring strategies for anti-photoaging is an important area of great interest.

Marine peptides are one of the richest sources of diverse bioactive compounds [5]. It was reported that mackerel (*Scomber japonicus*) muscle protein hydrolysates showed high antioxidant activities by evaluating the DPPH radical scavenging activity and SOD activity [6]. Peptides derived from peanut worms (*Sipunculus nudus*) showed high anti-inflammatory activity by inhibiting NO production of RAW 264.7 macrophages and reducing the expression of proinflammatory cytokine genes [7]. In the marine aquaculture industry, *Pinctada martensii* is a major economically valuable shellfish [8] and is distributed in South China, Japan, Australia, and Southeast Asia [9]. Studies have shown that peptides isolated from *Pinctada martensii* have the ability to heal wounds, inhibit ACE, and show anti-photoaging activity. For example, purified peptides from *Pinctada martensii* increased the proliferation of human skin fibroblasts and human immortalised keratinocyte cells, accelerated epithelialization, and regulated the collagen I/III ratio in vivo [10]. The pearl oyster meat protein hydrolysate exhibited an effective antihypertensive effect on SD rats [11]. In addition, according to Wu et al. [12], *Pinctada martensii* peptides protected mouse skin from photoaging by decreasing the thickness ratio of the dermis and epidermis and increasing the density of collagen fibers. However, the identification and the structure–activity relationship of the anti-photoaging peptides derived from *Pinctada martensii* were still unclear.

Therefore, in this study, the anti-photoaging activity and the mechanism of peptides derived from *Pinctada martensii* meat were investigated. Furthermore, molecular docking was used to study the structure–activity relationship, in which peptides interacted with collagenase (MMP-1) and matrix lysing enzyme (MMP-3). The result will significantly contribute to the study of the mechanism of anti-photoaging peptides from marine shellfish.

## 2. Results and Discussion

### 2.1. Anti-Photoaging Activity of Pinctada martensii Meat Hydrolysates (PME)

**Figure 1:**
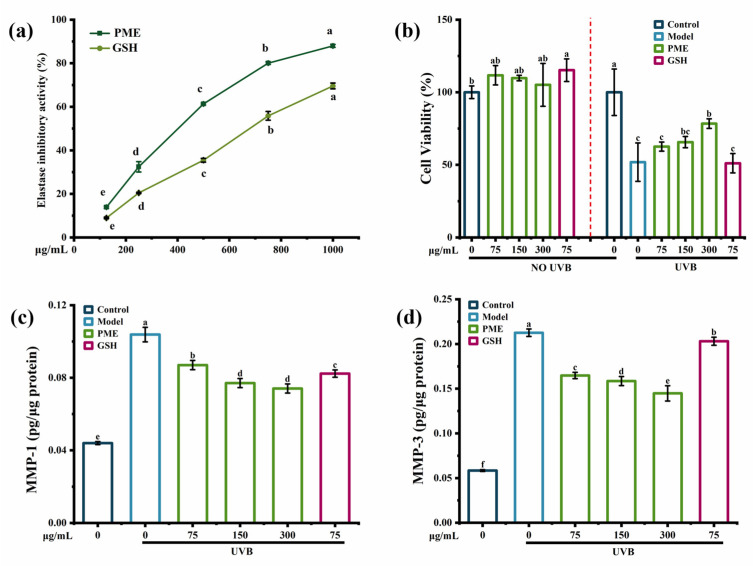
The anti-photoaging activity of PME. (**a**) Elastase inhibition activity of PME. (**b**) The effect of PME on UVB-induced cell viability in HaCaT cells. (**c**) The effect of PME on the content of MMP-1 in HaCaT cells. (**d**) The effect of PME on the content of MMP-3 in HaCaT cells. Different lowercase letters indicate significant differences at *p*  <  0.05. Data are presented as the mean ± SD of three independent experiments.

Elastase inhibition activity was used as an important mechanism to protect the skin from photoaging [13]. The elastase inhibition activity was significantly increased by *Pinctada martensii* meat hydrolysates (PME) in a dose-dependent manner (Figure 1a) and was up to 87.96% at 300 mg/mL of PME. In addition, the elastase inhibition activity of PME was better than that of the positive control group. Thus, PME might prevent skin elasticity loss. Peptides derived from bovine elastin hydrolysates [13] and the chia seed [14] also showed anti-photoaging ability with elastase inhibition activity, but the elastase inhibition was around 50–60%.

The MTT assay showed that there were no toxic effects of PME on HaCaT cells, and cell viability was decreased by 50% after UVB irradiation, while it was increased in a dose-dependent manner with PME treatment (Figure 1b). A previous study suggested that peptides obtained from oysters showed the anti-photoaging effect at a concentration of 100μg/mL, which improved the HaCaT cells’ viability from 61.49% after UVB irradiation to 84.99% [15]. Besides, UV irradiation would affect the contents of MMP-1 and MMP-3 by a complex cascade of transcription factors and protein kinases [16]. MMP-1 decomposed intact fibrillar collagen, and MMP-3 could further decompose collagen fragments that had already been damaged [17]. As a result, UVB irradiation increased the contents of MMP-1 and MMP-3 in HaCaT cells, which were 2.5-fold and 3.5-fold higher than those of the control group, respectively (Figure 1c,d). PME inhibited the contents of MMP-1 and MMP-3 in a dose-dependent manner. In addition, compared to the positive control group, PME showed a better effect on increasing cell viability and decreasing the contents of MMP-1 and MMP-3. Many reports have shown the inhibition of MMPs by peptides in a UVB-induced photoaging model. For example, peptides from abalone effectively reduced the secretion of MMP-1 in UVB-induced HaCaT cells in a dose-dependent manner [18]. Heptapeptide isolated from *Isochrysis zhanjiangensis* also reduced the secretions of MMP-1 and MMP-3 in HaCaT cells [19]. Therefore, all the data showed that PME could increase the elastase inhibition activity and cell viability while reducing the contents of MMP-1 and MMP-3 in UVB-induced HaCaT cells, indicating PME had potential anti-photoaging activity.

### 2.2. Screening of Fractions Separated by Ultrafiltration and Sephadex G-25 Gel Filtration Chromatography

**Figure 2:**
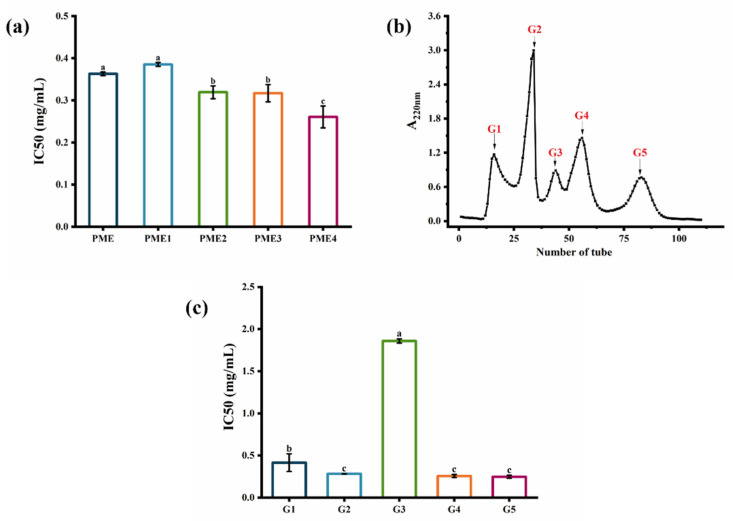
Separation and purification of PME by ultrafiltration and Sephadex G-25 gel filtration chromatography. (**a**) Elastase inhibition IC50 values of PME and ultrafiltered fractions: PME1 (>10 kDa), PME2 (5–10 kDa), PME3 (3–5 kDa), and PME4 (<3 kDa). (**b**) Elution profile on Sephadex G-25 gel filtration chromatography. (**c**) Elastase inhibition IC50 values of the fractions G1, G2, G3, G4, and G5. Different lowercase letters indicate significant differences at *p* < 0.05. Data are presented as the mean ± SD of three independent experiments.

PME was first separated by ultrafiltration. Four ultrafiltered fractions were obtained: PME1 (MW > 10 kDa), PME2 (5 kDa < MW < 10 kDa), PME3 (3 kDa < MW < 5 kDa), and PME4 (MW < 3 kDa). As shown in Figure 2a, PME4 (IC_50_ = 0.26 mg/mL) showed higher elastase inhibition activity than PME and other purified fractions. Similar to previous reports, ultrafiltration fractions with lower molecular weights showed higher elastase inhibition activity because they could enter the hydrophobic channel of elastase and inhibit the hydrolysis of elastin [20]. Thus, PME4, which had a lower molecular weight, might be able to enter the hydrophobic channel of elastase and showed higher elastase inhibition activity, and then it was considered to be further purified by Sephadex G-25 gel filtration chromatography. Five purified fractions, G1, G2, G3, G4, and G5, were obtained after purification by Sephadex G-25 gel filtration chromatography (Figure 2b). The elastase inhibition IC_50_ values of fractions G1, G2, G3, G4, and G5 were 0.41 mg/mL, 0.28 mg/mL, 1.86 mg/mL, 0.26 mg/mL, and 0.25 mg/mL (Figure 2c), respectively, indicating that the elastase inhibition activities of fractions G2, G4, and G5 were higher than those of fractions G1 and G3. As shown in Table 1, fraction G2 had a protein recovery rate (14.01%) that was six to eight times higher than fractions G4 and G5. Therefore, the fraction G2 was then used for further study. Previous studies indicated that a purified fraction of peptides with a molecular weight less than 3 kDa separated using a macroporous resin column had the strongest elastase inhibitory activity (65.80%) [13], and the purified fraction separated by size exclusion chromatography also showed the elastase inhibition activity (55.61%) [14]. Therefore, the ultrafiltered fraction with a molecular weight less than 3 kDa exhibited the highest elastase inhibitory activity, and then it could be separated into five purified fractions, while the purified fraction G2 had the potential for further study.

### 2.3. Anti-Photoaging Mechanism of the Fraction G2

#### 2.3.1. Effects of the Fraction G2 on Cell Viability, the Contents of MMP-1 and MMP-3

**Figure 3:**
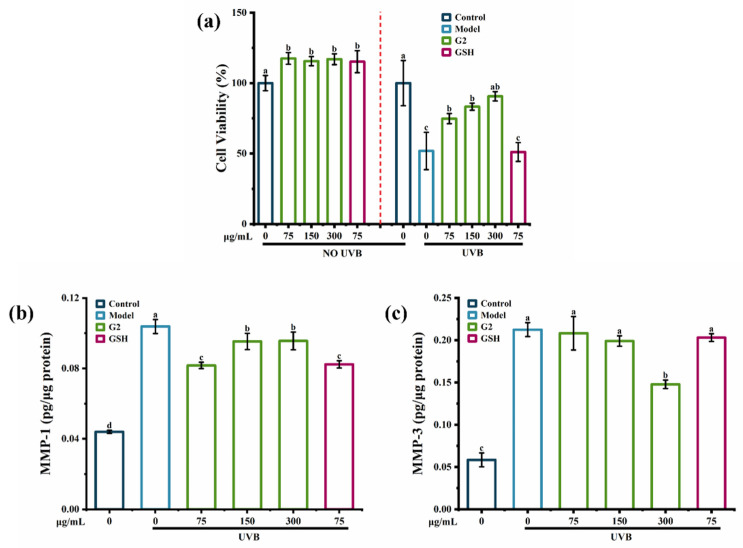
The anti-photoaging activity of the fraction G2. (**a**) The effect of the fraction G2 on UVB-induced cell viability in HaCaT cells. (**b**) The effect of the fraction G2 on the content of MMP-1 in HaCaT cells. (**c**) The effect of the fraction G2 on the content of MMP-3 in HaCaT cells. Different lowercase letters indicate significant differences at *p*  <  0.05. Data are presented as the mean ± SD of three independent experiments.

UVB entailed a complex network of signaling pathways, which were the final executors of UVB-induced cell death [21]. The MTT assay showed that there were no toxic effects of the fraction G2 on HaCaT cells. UVB irradiation significantly decreased cell viability by 50%, while the fraction G2 increased cell viability in a dose-dependent manner (Figure 3a). In addition, when the fraction G2 and GSH were both at 75 μg/mL, the fraction G2 was more effective than GSH at increasing cell viability. Compared with the control group, UVB irradiation caused a 2.5-fold increase in the content of MMP-1 (Figure 3b) and a 4.0-fold increase in the content of MMP-3 (Figure 3c). The fraction G2 significantly reduced the content of MMP-1 in HaCaT cells and showed the best inhibiting effect at 75 μg/mL. The content of MMP-3 was reduced by the fraction G2 in a dose-dependent manner, and there was a significant effect at 300 μg/mL. Purified peptides derived from tilapia skin gelatin [22] and cod skin gelatin hydrolysates [23] were previously shown to inhibit UVB-induced MMP-1 activity. Therefore, all the data confirmed that fraction G2 protected HaCaT cells against photoaging by increasing cell viability and decreasing contents of MMP-1 and MMP-3.

#### 2.3.2. Effects of the Fraction G2 on the Expression of p38, JNK, ERK, MMP-1, and MMP-3

To study the mechanism of anti-photoaging activity, the expression of *p38*, *JNK*, *ERK*, *MMP-1*, and *MMP-3* was analyzed. After the stimulation of UV, the generated free radicals stimulated the phosphorylation of extracellular signal-regulated kinase (ERK), c-Jun N-terminal kinase (JNK), and p38 MAPK through MAPK signaling pathway [24], which led to the photoaging.

**Figure 4:**
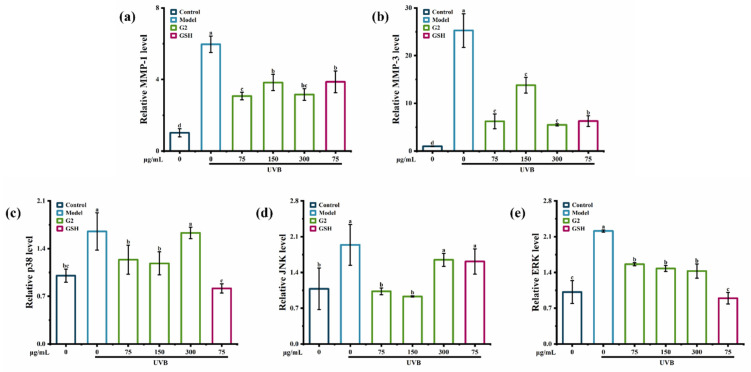
The effect of the fraction G2 on the relative expression of *MMP-1* (**a**), *MMP-3* (**b**), *p38* (**c**), *JNK* (**d**), and *ERK* (**e**) in HaCaT cells. Different lowercase letters indicate significant differences at *p* < 0.05. Data are presented as the mean ± SD of three independent experiments.

The mRNA levels of *p38*, *JNK*, *ERK*, *MMP-1*, and *MMP-3* in HaCaT cells were significantly upregulated by UVB irradiation. Compared with the control group, UVB irradiation significantly upregulated the mRNA levels of *MMP-1* and *MMP-3* in HaCaT cells by 6- and 25-fold, indicating that UVB irradiation activated the production of MMP-3 more than that of MMP-1, consistent with the result of the ELISA assay (Figure 4a,b). Fraction G2 suppressed the mRNA levels of *MMP-1* and *MMP-3* by 51.51% and 78.29%. In addition, the fraction G2 (75 μg/mL and 150 μg/mL) significantly suppressed the expression of *p38* and *JNK* (Figure 4c,d) and downregulated the expression of *ERK* in a dose-dependent manner (Figure 4e). Many studies have found anti-photoaging mechanisms in mRNA levels. For example, peptides derived from oyster hydrolysate [25] and purified peptides obtained from Astragalus membranaceus [26] significantly inhibited *MMP-1* and *MMP-3* expression in the photoaging cells model. *Ostrea rivularis* peptides suppressed the expression of p38, which was upregulated by UV irradiation [27]. Puerarin inhibited the expression of *p38*, *JNK*, and *ERK* in human fibroblasts [28]. Thus, the results confirmed that the fraction G2 showed anti-photoaging activity in HaCaT cells by regulating the mRNA levels of *p38*, *JNK*, *ERK*, *MMP-1*, and *MMP-3* in the MAPK signaling pathway.

### 2.4. Identification of Peptides in the Fraction G2

**Figure 5:**
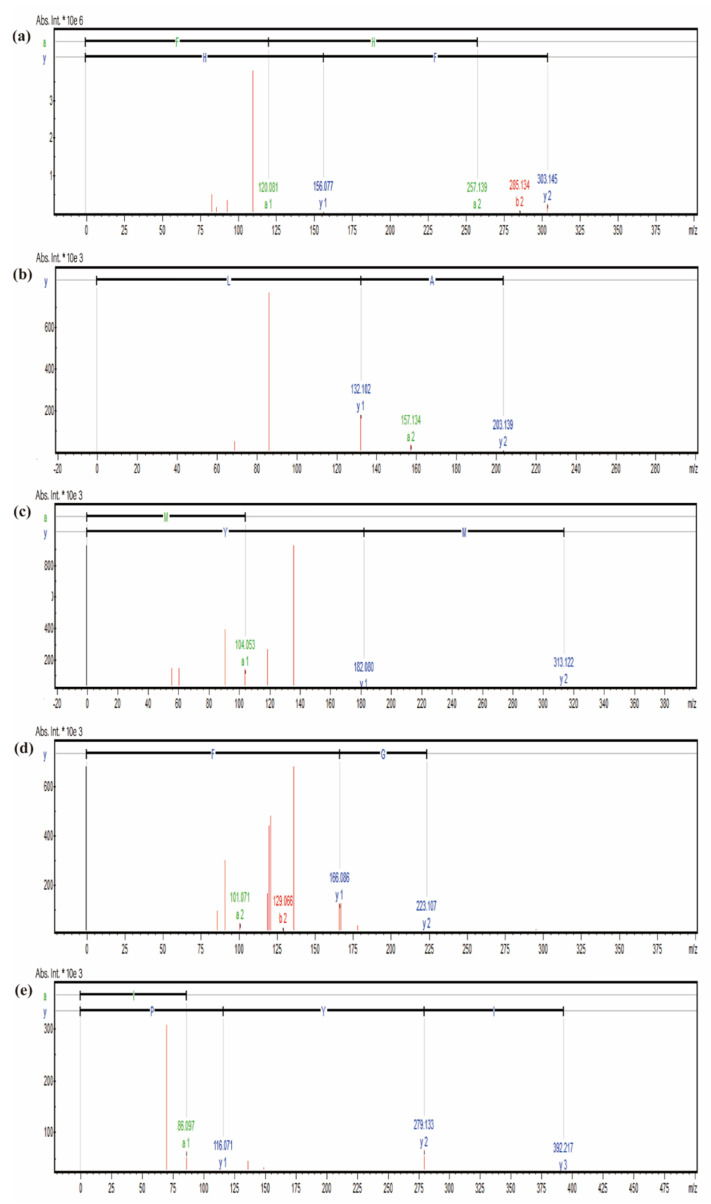
The mass spectra result of peptides FH (**a**), AL (**b**), MY (**c**), AGF (**d**), and IYP (**e**).

For the identification of peptides, 171 peptides were identified by UHPLC-MS/MS with a molecular weight range of 189.12 to 625.29 Da. The identified peptides were 2–7 amino acids, and peptides with 2–3 amino acids accounted for 74.85%, because short peptides might be obtained by gastrointestinal digestion [13]. Previous studies suggested that peptides with digestive stability showing the average chain length of 4.5 ± 2.0 amino acid residues and molecular weight of 547.78 ± 233.17 M [29], indicating that the fraction G2 might have digestive stability. In addition, it was reported that peptides could be effectively absorbed and distributed in the skin and can provide an effective effect on photoaging skin [24], indicating that the fraction G2 might maintain the anti-photoaging activity on photoaging skin after absorption through the gastrointestinal system. According to the peak areas, PeptideRanker (http://distilldeep.ucd.ie/PeptideRanker/, 22 March 2022) scores, and ToxinPred databases (https://webs.iiitd.edu.in/raghava/toxinpred, 22 March 2022), non-toxic peptides with higher peak areas and PeptideRanker scores (>0.4) were considered for synthesis (Table 2). Previous reports suggested that peptides with anti-photoaging activity contained more hydrophobic amino acids, especially the N-terminal amino acid alanine, such as Ala-Thr-Pro-Gly-Asp-Glu-Gly (ATPGDEG) [18] and Ala-Asp-Ile-Tyr-Thr-Glu-Glu-Ala-Gly-Arg (ADIYTEEAGR) [30]. Thus, peptide sequences Phe-His (FH), Ala-Leu (AL), Met-Tyr (MY), Ala-Gly-Phe (AGF), and Ile-Tyr-Pro (IYP) were considered to be synthesized by solid-phase synthesis (Figure 5a–e).

### 2.5. Anti-Photoaging Activity of Synthesized Peptides

#### 2.5.1. Effects of Synthesized Peptides on Cell Viability

**Figure 6:**
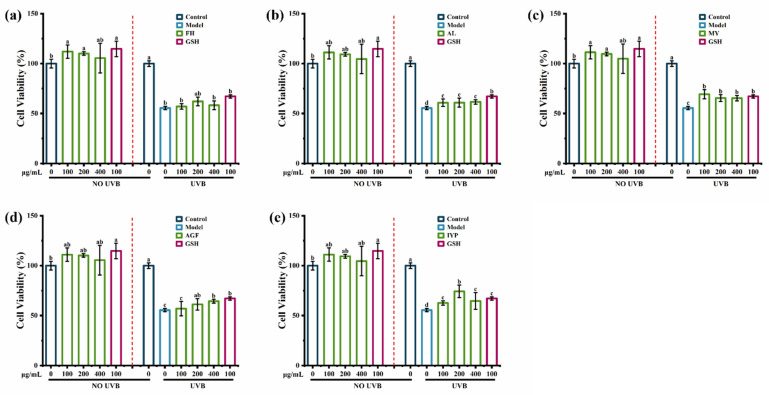
The effect of synthesized peptides FH (**a**), AL (**b**), MY (**c**), AGF (**d**), and IYP (**e**) on UVB-induced cell viability in HaCaT cells. Different lowercase letters indicate significant differences at *p* < 0.05. Data are presented as the mean ± SD of three independent experiment.

The results suggested that there were no toxic effects of synthesized peptides FH, AL, MY, AGF, and IYP on HaCaT cells. As shown in Figure 6b–e, synthesized peptides AL, MY, AGF, and IYP significantly increased the cell viability compared with the model group, while there was no significant effect of FH (Figure 6a).

Further, the synthesized peptide AGF increased the cell viability in a dose-dependent manner. A previous study showed that hydrophobic amino acids had a substantial impact on anti-photoaging activity [18], and hydrophobic amino acids in collagen-derived peptides were reported to enhance radical scavenging activity and exhibit antioxidant activity, which was closely related to anti-photoaging activity [31]. Hydrophobic amino acids accounted for 50% of FH and MY, while accounting for 66% of the synthesized peptides AL, AGF, and IYP. Besides, the phenolic hydroxyl of tyrosine could directly act as hydrogen donors to capture free radicals, thereby achieving the effect of scavenging free radicals, which would induce photoaging [32], so MY also significantly increased cell viability. Therefore, FH had less impact on cell viability. Many reports have shown the cell viability protecting effects of peptides. For example, a previous study suggested that the synthesized peptides Ala-Thr-Pro-Gly-Asp-Glu-Gly (ATPGDEG) [18], Thr-Ala-Leu-Ala-Ile-Asp-Ala-Ile-Ile-Asn-Gln-Lys (TALAIDAIINQK), and Val-Leu-Val-Pro-Thr-Gln-Glu-Ala-Val-Gln-Lys (VLVPTQEAVQK) from *Crassostrea Hongkongensis* [33] also increased the cell viability of HaCaT cells damaged by UVB irradiation. Therefore, the synthesized peptides AL, MY, AGF, and IYP showed better effects on increasing cell viability than FH.

#### 2.5.2. Effects of Synthesized Peptides on the Content of MMP-1 and MMP-3

**Figure 7:**
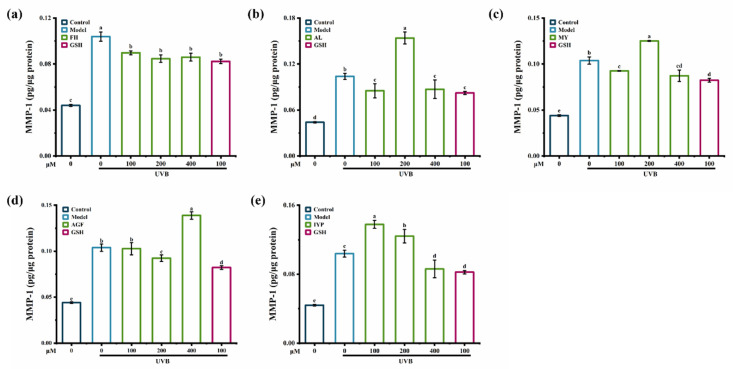
The effect of synthesized peptides FH (**a**), AL (**b**), MY (**c**), AGF (**d**) and IYP (**e**) on the content of MMP-1 in HaCaT cells. Different lowercase letters indicate significant differences at *p* < 0.05. Data are presented as the mean ± SD of three independent experiments.

Compared to synthesized peptides AGF and IYP, synthesized peptides FH, AL, and MY exhibited a stronger inhibitory effect on the content of MMP-1 (Figure 7).

FH (100–400 μM) significantly reduced the content of MMP-1 caused by UVB irradiation (Figure 7a), while MY and AL showed similar inhibitory effects both at 100 μM and 400 μM (Figure 7b,c). Thus, synthesized peptide FH had the strongest inhibitory effect on the content of MMP-1. The reason might be that the synthesized peptide FH was composed of phenylalanine and histidine, and phenylalanine had an aromatic radical that absorbed ultraviolet irradiation and then constituted the natural moisturizing factor layer, contributing to skin protection [34]. A previous study showed that Gly-Phe-Ser-Gly-Leu-Asp-Gly-Ala-Lys-Gly-Asp (GFSGLDGAKGD) [23], which contained phenylalanine, also showed an inhibitory effect on MMP-1.

**Figure 8:**
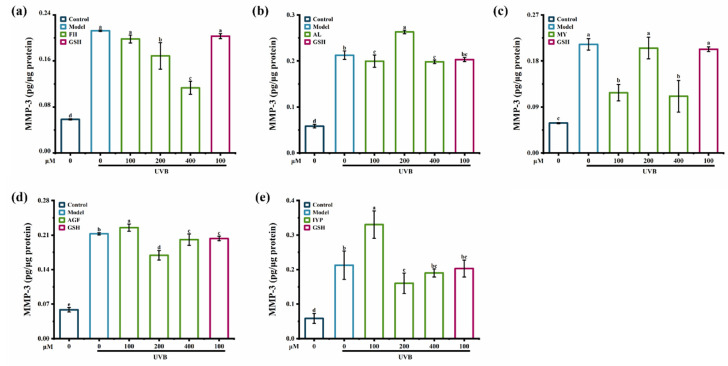
The effect of synthesized peptides FH (**a**), AL (**b**), MY (**c**), AGF (**d**), and IYP (**e**) on the content of MMP-3 in HaCaT cells. Different lowercase letters indicate significant differences at *p* < 0.05. Data are presented as the mean ± SD of three independent experiments.

As shown in Figure 8, compared with synthesized peptides MY, AL, AGF, and IYP, the synthesized peptide FH showed a stronger inhibitory effect on the content of MMP-3 in HaCaT cells. FH (100–400 μM) reduced the content of MMP-3 in a dose-dependent manner, and by 52% at 400 μM compared with the model group (Figure 8a). Treatment with the synthesized peptide MY (100 μM and 400 μM) significantly reduced the content of MMP-3, whereas there was no inhibitory effect of MY at 200 μM (Figure 8c). It was reported that Leu-Ser-Gly-Tyr-Gly-Pro (LSGYGP) also significantly inhibited the content of MMP-3 [35].

The synthesized peptide FH had no significant effects on cell viability compared to other synthesized peptides, but it showed inhibitory effects on the contents of MMP-1 and MMP-3. The activities of peptides are highly dependent on the chain length, molecular weight, and molecular interactions of the peptides [36]. This suggested that the interaction between the synthesized peptide FH and MMP-1 or MMP-3 might be the reason for the result. To further explore the interaction between FH and MMPs, FH was chosen to perform the molecular docking analysis. Moreover, synthetic peptides identified in the fraction G2 showed anti-photoaging activity, suggesting that they might have contributed to the anti-photoaging activity of the fraction G2.

### 2.6. Molecular Docking Analysis of FH with MMP-1 and MMP-3

**Figure 9:**
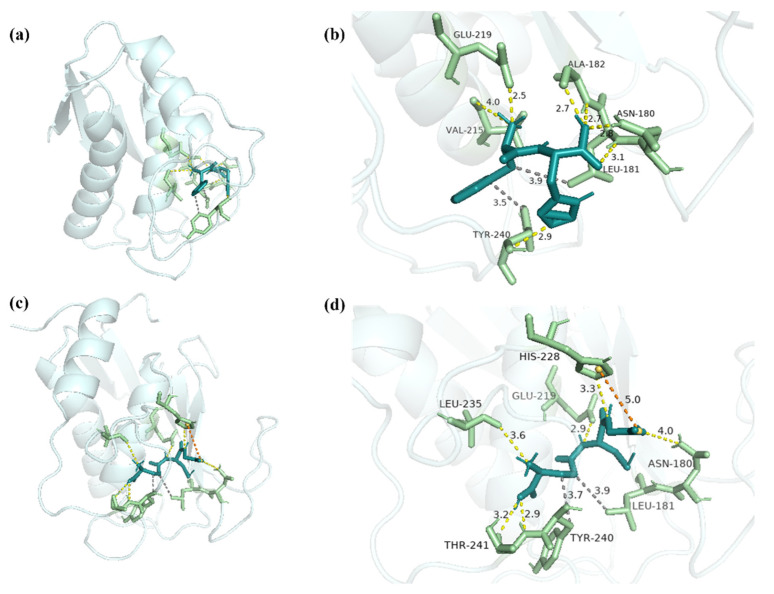
The docking interactions of MMP-1 with FH and GSH. (**a**) The three-dimensional structure of FH-MMP-1 complex. (**b**) The interaction details between FH and MMP-1. (**c**) The three-dimensional structure of GSH-MMP-1 complex. (**d**) The interaction details between GSH and MMP-1. The dashed yellow line represents the hydrogen bonds, the dashed orange line represents salt bridge, and the dashed gray line represents hydrophobic interaction.

Given the activities of the synthesized peptide FH, potential binding sites between biomacromolecules and peptides were analyzed by molecular docking. Hydrophobic interactions and electrostatic interactions, which include hetero-atom-hydrogen bonds, salt bridges, and van der Waals interactions (π–π interactions), are required for protein–ligand complex formation and stability [37]. Besides, hydrogen bonds not only mediate protein–ligand binding but also influence the physicochemical properties of the molecules, which play an important role [38]. Due to the deep cavity that existed in the active site of MMP-1 [39], FH was able to interact with MMP-1 (−7.63 kcal/mol) (Figure 9a). Seven hydrogen bonds with lengths of 2.8, 3.1, 2.7, 2.7, 4.0, 2.5, and 2.9 were shown between FH and the residues Asn180, Leu181, Ala182, Val215, Glu219, and Tyr240 (Figure 9b).

By hydrophobic interactions, the residues Leu181 and Tyr240 were bonded with FH. In the positive group, GSH and MMP-1 combined to form a complex with an interaction energy of −3.86 kcal/mol (Figure 9c). Six hydrogen bonds were discovered in the complex, combining GSH and Asn180, Glu219, His228, Leu235, and Thr241 residues with lengths of 4.0, 2.9, 3.3, 3.6, 2.9, and 3.2 (Figure 9d). Residues Leu181, Tyr240, and GSH interacted hydrophobically, and the complex formed a salt bridge with residue His228 (Table 3). Previous studies demonstrated that Gly-Tyr-Thr-Gly-Leu (GYTGL) interacted with MMP-1 by residues Gly179, Asn180, Leu181, Ala182, Glu219, Tyr240, and Zn265 [40]. Therefore, Asn180, Leu181, Ala182, Glu219, and Tyr240 played important roles in the combination of the ligand and MMP-1. It was reported that epigallocatechin gallate (EGCG) was also combined with MMP-1 as an inhibitor by hydrophobic interaction [41]. The results suggested that the high inhibitory activity of FH on MMP-1 might be mediated by the formation of hydrogen bonds and hydrophobic interaction at the binding site.

**Figure 10:**
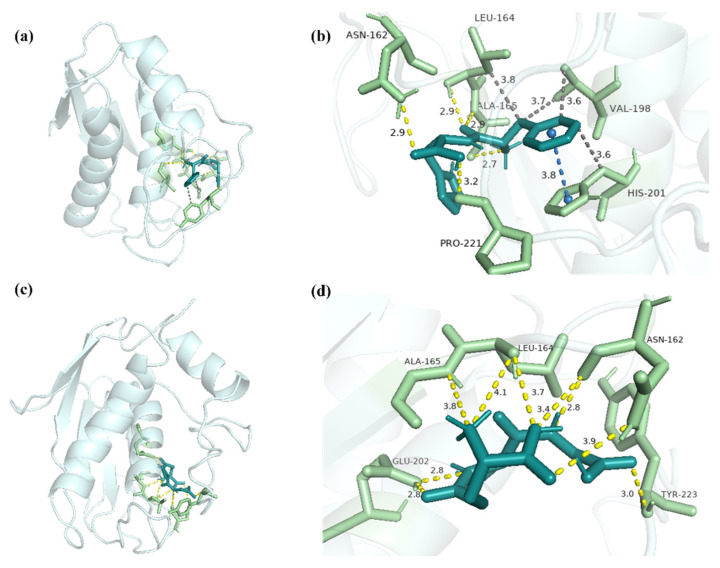
The docking interactions of MMP-3 with FH and GSH. (**a**) The three-dimensional structure of FH-MMP-3 complex. (**b**) The interaction details between FH and MMP-3. (**c**) The three-dimensional structure of GSH-MMP-3 complex. (**d**) The interaction details between GSH and MMP-3. The dashed yellow line represents the hydrogen bonds, the dashed orange line represents salt bridge, and the dashed gray line represents hydrophobic interaction.

FH and MMP-3 combined to form the complex, which had a binding energy of -7.31 kcal/mol (Figure 10a). Residues Asn162, Leu164, Ala165, and Pro221 contributed to the interaction between FH and MMP-3, forming six hydrogen bonds with lengths of 2.9, 2.7, 2.9, 2.9 and 3.2 (Figure 10b). FH combined with the residues Leu164, Val198, and His201, exhibiting the hydrophobic interaction (Table 4). With the residue His201, π-stacking was created in the complex of FH-MMP-1. GSH and MMP-3 combined to form a complex with an interaction energy of -4.82 kcal/mol (Figure 10c). Nine hydrogen bonds, with lengths of 3.4, 2.8, 3.9, 4.1, 3.8, 3.8, 2.8, 2.8, and 3.0, were formed between GSH and MMP-3 through residues Asn162, Leu164, Ala165, Glu202, and Tyr223 (Figure 10d). Previous studies showed that the glyceroglycolipid combined with MMP-3 by residues Asn162, Leu164, His201, His211, and Tyr223 [42]. 

The results suggested that residues Asn162 and Leu164 were essential in the interaction between the ligand and MMP-3. It was reported that hydrophobic interaction was also important in the interaction between the ligand and MMP-3 in the Rutin study [43]. Therefore, the high inhibitory activity of FH on MMP-3 might be caused by the formation of hydrogen bonds, hydrophobic interaction, and π-stacking at the binding site.

## 3. Materials and Methods

### 3.1. Chemicals and Materials

*Pinctada martensii* meat was obtained from Zhanjiang Ronghui Pearl Co., Ltd. (Zhanjiang, China). Pepsin (400 U/mg), and N-Succinyle-Ala-Ala-Ala-pNA was purchased from Sigma-Aldrich (Shanghai, China). Elastase (30 U/mg) and trypsin (250 U/mg) were purchased from Yuanye Bio-Technology Co., Ltd. (Shanghai, China). Chymotrypsin (800 U/mg) was purchased from Macklin Biochemical Co., Ltd. (Shanghai, China). Human immortalized epidermal cells HaCaT (PUMC000373) were obtained from the Institute of Basic Medicine, Chinese Academy of Medical Sciences (Beijing, China). Dulbecco’s Modified Eagle’s Medium (DMEM), fetal bovine serum (FBS), penicillin–streptomycin, trypsin-ethylenediaminetetraacetic acid, and phosphate-buffered saline (PBS) were acquired from Gibco Biotechnology Co., Ltd. (Grand Island, NY, USA). Human MMP-1 (Total) ELISA kit and Human MMP-3 (Total) ELISA kit were purchased from Neobioscience Technology Co., Ltd. (Guangzhou, China). The bicinchoninic acid (BCA) protein quantitation assay kit was purchased from KeyGenBio (Nanjing, China). Other reagents were analytical grade.

### 3.2. Enzymatic Hydrolysis of Pinctada Martensii Meat

*Pinctada martensii* meat was hydrolyzed according to the reported method [44]. *Pinctada martensii* meat (100 g) was chopped and homogenized with 200 mL of distilled water by the meat grinder (BL 18PP, Guangzhou Telong Instrument Co. Ltd., Guangzhou, China), and the mixture was then adjusted to pH 3 by adding 1 M HCl. The reaction was carried out at 37 °C with constant shaking for 2 h by the water bath shaker (SHA-CA, Changzhou Xiuhua Instrument Co. Ltd., Changzhou, China) after the addition of pepsin at 2000 U/mL. Pepsin was then inactivated by adjusting pH to 7.5 with 1 M NaOH. Next, 100 U/mL of trypsin and 25 U/mL of chymotrypsin were added, and the reaction was kept for 2 h at 37 °C with constant shaking. The reaction mixture was heated in boiling water for 10 min to inactivate enzymes. After centrifuging (H1850, XiangYi Centrifuge Instrument Co., Ltd., Changsha, China) at 8000 r/min for 20 min, the supernatant was collected and freeze-dried using a freeze-dryer (ALPHA 1–2 LDplus, Christ, Osterode, Germany) to obtain the enzymatic hydrolysate of *Pinctada martensii* meat (PME).

### 3.3. Separation and Purification of PME

PME was fractionated by ultrafiltration (VF20P0, Sartorius Stedim UK Ltd., Epsom, UK), with molecular weight (MW) cut-off membranes of 10 kDa, 5 kDa, and 3 kDa, resulting in four ultrafiltered fractions: PME1 (MW > 10 kDa), PME2 (5 kDa < MW < 10 kDa), PME3 (3 kDa < MW < 5 kDa), and PME4 (MW < 3 kDa). For further separation, the ultrafiltered fraction with the greatest elastase inhibition activity was purified by the Sephadex G-25 gel filtration column (4.6 × 60 cm). Distilled water was used as the mobile phase and flowed at a rate of 1.2 mL/min. The fractions containing the peptides were collected at 4 min intervals using an automatic fraction collector (BSZ-160, Shanghai Huxi Machinery Co. Ltd., Shanghai, China), and then were collected based on the UV absorbance, which was measured at 220 nm by the UV spectrophotometer (UV755B, Shanghai Youke Instrument Co. Ltd., Shanghai, China). The fractions separated by the Sephadex G-25 gel filtration column (G1, G2, G3, G4, and G5) were obtained, and the protein recovery rates of purified fractions were measured by the BCA assay kit.

### 3.4. Elastase Inhibition Activity Assay

The elastase inhibition activity was determined according to the method previously described, with some modifications [13]. Briefly, the reaction was carried out in 100 mM Tris-HCl (pH 8.0). Test samples (20 µL) were mixed with 100 µL of elastase at 100 U/mL, and then the mixture was incubated for 20 min at 37 °C. Next, N-(methoxysuccinyl)-Ala-Ala-Pro-Val p-nitroanilide substrate (80 µL, 2 mM) was added to the mixture and incubated for 20 min at 37 °C. Glutathione (GSH) was used as the positive control. Finally, the absorbance of the mixture was recorded at 410 nm by a microplate reader (SpectraMAX250, Molecular Devices, CA, USA). The elastase inhibition activity was calculated with Equation (1), where OD_control_ and OD_sample_ represented the optical density of the control group and sample group, respectively.

(1) $$\mathrm{Elastase}\;\mathrm{inhibition}\;\left(\%\right)=\frac{{\mathrm{OD}}_{\mathrm{control}}\;{-\;\mathrm{OD}}_{\mathrm{sample}}}{{\mathrm{OD}}_{\mathrm{control}}},$$

### 3.5. Cell Culture and UVB Irradiation

HaCaT cells were cultured in DMEM medium supplemented with 10% FBS and 1% penicillin-streptomycin at 37 °C with 5% CO_2_. Cells were washed with PBS before UVB irradiation. Afterward, cells were maintained in PBS-filled wells and exposed to 15 mJ/cm^2^ of UVB irradiation from a UVB lamp (UVB-313 NM, 8 W, Shenzhen Guanhongrui Instrument Co. Ltd., Shenzhen, China). Next, PBS was removed, and the DMEM medium containing 10% FBS and 1% penicillin-streptomycin was added to incubate at 37 °C with 5% CO_2_. 

### 3.6. Cell Viability Assay

HaCaT cells (1.5 × 10^4^ cells/well) were seeded in 96-well plates and incubated for 24 h. Then, cells were treated with GSH and peptides (PME, fraction G2, and synthesized peptides), which were prepared with serum-free media, and then the cells were incubated for 6 h. Concentrations of samples were selected on the basis of previous experimental experience. Concentrations of PME and G2 were 75–300 μg/mL, and the concentration of synthesized peptides was 100–400 μM. After UVB irradiation, as described in Section 3.5, cells were incubated for 24 h in DMEM medium supplemented with 10% FBS and 1% penicillin-streptomycin. Cells in each group were exposed to UVB irradiation, except for cells in the control group. Cell viability was measured by 3-[4,5-dimethylthiazol-2-yl]-2,5-diphenyltetrazolium bromide (MTT) assay according to the manufacturer’s procedure. GSH was used as a positive control.

### 3.7. Measurement of the Contents of MMP-1 and MMP-3

In 6-well plates, HaCaT cells (4.5 × 10^5^ cells/well) were seeded and incubated for 24 h. Then, cells were treated with GSH and peptides (PME, fraction G2, and synthesized peptides), which were prepared with serum-free media, and then the cells were incubated for 6 h. The concentrations of PME and G2 were 75–300 μg/mL, and the concentration of synthesized peptides was 100–400 μM. After UVB irradiation, as described in Section 3.5, cells were incubated for 24 h in DMEM medium supplemented with 10% FBS and 1% penicillin-streptomycin. Cells in each group were exposed to UVB irradiation except for cells in the control group. The ELISA kit was used to measure the contents of MMP-1 and MMP-3, and all values were normalized against total protein measured by the BCA assay. GSH was used as a positive control.

### 3.8. Quantitative Reverse Transcription-Polymerase Chain Reaction (qRT-PCR) Analysis

Total RNA was extracted from HaCaT cells using Trizol (Thermo-Fisher, Grand Island, NY, USA), and cDNA was synthesized using RevertAid First Strand cDNA Synthesis Kit (Thermo-Fisher, Grand Island, NY, USA), according to the manufacturer’s protocol. Next, RT-qPCR was performed by SYBR Green Master qPCR Mix (TSINGKE, Beijing, China), and the primers (Sangon Biotech, Shanghai, China) were shown in Table 5, which were designed by Oligo 7 software (Molecular Biology Insight, Inc., Cascade, CO, USA). Changes in gene expression were analyzed using the CFX48TM Real-Time System (Bio-Rad Laboratories, Inc., Hercules, CA, USA), and fold changes were calculated using 2^-ΔΔCT^ method, normalized with GADPH as the housekeeping gene.

### 3.9. Identification of Peptides by UHPLC-MS/MS

Peptides were identified by the ultra-high-performance liquid chromatography tandem mass spectrometry (UHPLC-MS/MS) system (Ultimate 3000/Q-Exactive, Thermo Fisher Scientific, San Jose, CA, USA). Samples (1 μL) were injected into the Accucore RP-MS column (2.6 μm, 100 × 2.1 mm) and were separated at a flow rate of 0.05 mL/min. The column temperature was 30–60 °C. The mobile phase consisted of 0.1% (*v*/*v*) formic acid in water (phase A) and acetonitrile (phase B), while the gradient elution was as follows: 0–4.00 min, 5.0% B; 4.00–6.00 min, 5.0–10.0% B; 6.00–30.00 min, 10.0–40.0% B; 30.00–34.00 min, 40.0–90.0% B; 34.00–40.00 min, 90% B; 40.00–42.00 min, 90.0–5.0% B; 42.00–52.00 min, 5.0% B. All the data were collected using the following parameters: resolution = 35,000, scan range = 100–1500 m/z, TopN = 4, stepped collision energy (20, 40, 60), and positive ion mode. The sequences of identified peptides were obtained by PepOS 1.0 software (Wuyi University, Guangzhou, China, 2022), and peptides with lengths ranging from 2 to 25 were identified.

### 3.10. Peptide Synthesis

The peptides were synthesized by Synpeptide Co., Ltd. (Nanjing, China). The purity of the synthesized peptides Phe-His (FH), Ala-Leu (AL), Met-Tyr (MY), Ala-Gly-Phe (AGF), and Ile-Tyr-Pro (IYP) was more than 95%.

### 3.11. Molecular Docking Analysis with FH

The receptor structures of MMP-1 (PDB ID: 966c) and MMP-3 (PDB ID: 2JT6) were available from the RCSB Protein Data Bank (https://www.rcsb.org/, 14 July 2022). Before molecular docking, the structures of the receptor were modified by removing water molecules, deleting the original ligand, and adding hydrogen atoms. The active center of MMP-1 was defined with coordinates X: 6.512, Y: -8.928, and Z: 35.713, while coordinates X: 8.152, Y: -1.725, and Z: 5.956 were performed for MMP-3. The synthesized peptide FH was used as the ligand, and the 3D structure was obtained by YINFO TECHNOLOGY Co., Ltd. (Guangzhou, China) (https://cloud.yinfotek.com/console/tools, 14 July 2022), while the structure of positive control GSH was obtained from PubChem (https://pubchem.ncbi.nlm.nih.gov/, 14 July 2022). Then, energy minimizing was used for the ligand pretreatment process. Energy values were calculated using AutoDock Tools Version 1.5.6 while performing molecular docking. Hydrogen bonds, hydrophobic interactions, π-staking, salt bridges, and the distance were calculated by the Protein-Ligand Interaction Profiler (https://plip-tool.biotec.tu-dresden.de/plip-web/plip/index, 16 July 2022). The docked positions and their interactions were visualized by Pymol 2.5 software (PymoL 2.5, Schrödinger Inc., New York, NY, USA).

### 3.12. Statistical Analysis

All the data were performed with at least three technical and three biological replicates, showing as mean ± standard deviation (SD). Statistical comparisons between different groups were performed using the one-way analysis of variance (ANOVA) followed by Tukey’s test. Differences were considered significant at a *p* value of 0.05 or less. All statistical analyses were performed using the SPSS 26 software (SPSS, Chicago, IL, USA).

## 4. Conclusions

In this study, we demonstrated that peptides from *Pinctada martensii* meat effectively reduced the UVB-induced photoaging in HaCaT cells. The effects were mainly due to promoting elastase inhibition activity and cell viability, reducing the contents of interstitial collagenase (MMP-1) and matrix lysing enzyme (MMP-3), and downregulating the expression of MMPs in HaCaT cells. In addition, the synthesized peptide Phe-His (FH) effectively combined with MMP-1 and MMP-3 to exhibit anti-photoaging activities. Thus, peptides from *Pinctada martensii* meat had the potential to inhibit photoaging. In future studies, we will focus on the anti-photoaging mechanism of synthesized peptides.

## Figures and Tables

**Table 1:** Protein recovery rates of purified fractions.

Purified Fractions	Protein Recovery Rates (%)
G1	21.47 ± 0.89 ^a^
G2	14.01 ± 1.86 ^b^
G3	21.15 ± 0.22 ^a^
G4	2.30 ± 0.20 ^c^
G5	1.68 ± 0.30 ^c^

Different lowercase letters in the same column mean significantly different (*p* < 0.05).

**Table 2:** Identification of peptides in the fraction G2.

Sequence	PeptideRanker Score	Length	Mass (Da)	ToxinPred
Phe-His (FH)	0.95283	2	303.14	Non-Toxin
Ala-Leu (AL)	0.4378	2	203.13	Non-Toxin
Met-Tyr (MY)	0.84347	2	392.21	Non-Toxin
Ala-Gly-Phe (AGF)	0.9568	3	294.14	Non-Toxin
Ile-Tyr-Pro (IYP)	0.57726	3	303.14	Non-Toxin

**Table 3:** The residues of interactions between MMP-1 and ligands.

	FH	GSH
Hydrophobic interaction	Leu181, Tyr240	Leu181, Tyr240
Hydrogen bonds	Asn180, Leu181, Ala182, VAL215, Glu219, Tyr240	Asn180, Glu219, His228, Leu235, Thr241
Salt bridges	-	His228
π-stacking	-	-

Note: No interaction with the key amino acid residue.

**Table 4:** The residues of interactions between MMP-3 and ligands.

	FH	GSH
Hydrophobic interaction	Leu164, Val198, His201	-
Hydrogen bonds	Asn162, Leu164, Ala165, Pro221	Asn162, Leu164, Ala165, Glu202, Tyr223
Salt bridges	-	-
π-stacking	His201	-

Note: No interaction with the key amino acid residue.

**Table 5:** Primer sequences of genes in qRT-PCR.

Gene	Forward Primer (5′-3′)	Reverse Primer (3′-5′)
*MMP-1*	GATGTGGAGTGCCTGATGTG	TGCTTGACCCTCAGAGACCT
*MMP-3*	CACTCACAGACCTGACTCGG	GAGTCAGGGGGAGGTCCATA
*p38*	ATGCCAAGCCATGAGGCAA	GCATCTTCTCCAGCAAGTCG
*ERK*	GCCGAAGCACCATTCAAGTT	CCTCTGAGCCCTTGTCCTGA
*JNK*	CAGCCCTCTCCTTTAGGTGC	GCTGCTGCTTCTAGACTGCT
*GAPDH*	TCCACTGGCGTCTTCACCACCAT	GGAGGCATTGCTGATGATCTTGAGG

## Data Availability

Data are available in a publicly accessible repository.

## References

[1] Petruk G., Del Giudice R., Rigano M.M., Monti D.M. (2018). Antioxidants from Plants Protect against Skin Photoaging. Oxid. Med. Cell. Longev..

[2] Wang P.W., Hung Y.C., Lin T.Y., Fang J.Y., Yang P.M., Chen M.H., Pan T.L. (2019). Comparison of the Biological Impact of UVA and UVB upon the Skin with Functional Proteomics and Immunohistochemistry. Antioxidants.

[3] Yao W., Chen X., Li X., Chang S., Zhao M., You L. (2021). Current trends in the anti-photoaging activities and mechanisms of dietary non-starch polysaccharides from natural resources. Crit. Rev. Food Sci. Nutr..

[4] Fanjul-Fernandez M., Folgueras A.R., Cabrera S., Lopez-Otin C. (2010). Matrix metalloproteinases: Evolution, gene regulation and functional analysis in mouse models. Biochim. Biophys. Acta.

[5] Kandi S., Baskaran Stephen I., Bing-Huei C. (2021). Recent developments on production, purification and biological activity of marine peptides. Food Res. Int..

[6] Bashir K.M.I., Sohn J.H., Kim J.S., Choi J.S. (2020). Identification and characterization of novel antioxidant peptides from mackerel (*Scomber japonicus*) muscle protein hydrolysates. Food Chem..

[7] Sangtanoo P., Srimongkol P., Saisavoey T., Reamtong O., Karnchanatat A. (2020). Anti-inflammatory action of two novel peptides derived from peanut worms (*Sipunculus nudus*) in lipopolysaccharide-induced RAW264.7 macrophages. Food Funct..

[8] Ma Y., Wu Y., Li L. (2018). Relationship between primary structure or spatial conformation and functional activity of antioxidant peptides from *Pinctada fucata*. Food Chem..

[9] Hao R., Du X., Yang C., Deng Y., Zheng Z., Wang Q. (2019). Integrated application of transcriptomics and metabolomics provides insights into unsynchronized growth in pearl oyster *Pinctada fucata martensii*. Sci. Total Environ..

[10] Zhang T., Yang F., Qin X., Yang X., Zhang C., Wan Z., Lin H. (2022). Investigation of the In Vivo, In Vitro, and In Silico Wound Healing Potential of *Pinctada martensii* Purified Peptides. Mar. Drugs.

[11] Liu P., Lan X., Yaseen M., Wu S., Feng X., Zhou L., Sun J., Liao A., Liao D., Sun L. (2019). Purification, Characterization and Evaluation of Inhibitory Mechanism of ACE Inhibitory Peptides from Pearl Oyster (*Pinctada fucata martensii*) Meat Protein Hydrolysate. Mar. Drugs.

[12] Yanyan W., Qian T., Laihao L., Muhammad Naseem K., Xianqing Y., Zhongmin Z., Xiao H., Shengjun C. (2013). Inhibitory effect of antioxidant peptides derived from *Pinctada fucata* protein on ultraviolet-induced photoaging in mice. J. Funct. Foods.

[13] Liu Y., Zheng L., Xu J.C., Sun-Waterhouse D., Sun B.G., Su G.W., Zhao M.M. (2020). Identification of novel peptides with high stability against in vitro hydrolysis from bovine elastin hydrolysates and evaluation of their elastase inhibitory activity. Int. J. Food Sci. Technol..

[14] Aguilar-Toala J.E., Liceaga A.M. (2020). Identification of chia seed (*Salvia hispanica* L.) peptides with enzyme inhibition activity towards skin-aging enzymes. Amino Acids.

[15] Peng Z., Gao J., Su W., Cao W., Zhu G., Qin X., Zhang C., Qi Y. (2022). Purification and Identification of Peptides from Oyster (*Crassostrea hongkongensis*) Protein Enzymatic Hydrolysates and Their Anti-Skin Photoaging Effects on UVB-Irradiated HaCaT Cells. Mar. Drugs.

[16] Garg C., Sharma H., Garg M. (2020). Skin photo-protection with phytochemicals against photo-oxidative stress, photo-carcinogenesis, signal transduction pathways and extracellular matrix remodeling—An overview. Ageing Res. Rev..

[17] Gu Y., Han J., Jiang C., Zhang Y. (2020). Biomarkers, oxidative stress and autophagy in skin aging. Ageing Res. Rev..

[18] Chen J., Liang P., Xiao Z., Chen M.-F., Gong F., Li C., Zhou C., Hong P., Jung W.-K., Qian Z.-J. (2019). Antiphotoaging effect of boiled abalone residual peptide ATPGDEG on UVB-induced keratinocyte HaCaT cells. Food Nutr. Res..

[19] Zheng Z., Xiao Z., He Y.-L., Tang Y., Li L., Zhou C., Hong P., Luo H., Qian Z.-J. (2021). Heptapeptide Isolated from Isochrysis zhanjiangensis Exhibited Anti-Photoaging Potential via MAPK/AP-1/MMP Pathway and Anti-Apoptosis in UVB-Irradiated HaCaT Cells. Mar. Drugs.

[20] Liu Y., Su G., Zhou F., Zhang J., Zheng L., Zhao M. (2018). Protective Effect of Bovine Elastin Peptides against Photoaging in Mice and Identification of Novel Antiphotoaging Peptides. J. Agric. Food Chem..

[21] Van Laethem A., Van Kelst S., Lippens S., Declercq W., Vandenabeele P., Janssens S., Vandenheede J.R., Garmyn M., Agostinis P. (2004). Activation of p38 MAPK is required for Bax translocation to mitochondria, cytochrome c release and apoptosis induced by UVB irradiation in human keratinocytes. FASEB J..

[22] Xiao Z., Liang P., Chen J., Chen M.F., Gong F., Li C., Zhou C., Hong P., Yang P., Qian Z.J. (2019). A Peptide YGDEY from Tilapia Gelatin Hydrolysates Inhibits UVB-mediated Skin Photoaging by Regulating MMP-1 and MMP-9 Expression in HaCaT Cells. Photochem. Photobiol..

[23] Jiaohan L., Hu H., Yan F., Tingting Y., Bafang L. (2017). Identification of MMP-1 inhibitory peptides from cod skin gelatin hydrolysates and the inhibition mechanism by MAPK signaling pathway. J. Funct. Foods.

[24] Chongyang L., Yu F., Hongjie D., Qiang W., Ruichang G., Yuhao Z. (2022). Recent progress in preventive effect of collagen peptides on photoaging skin and action mechanism. Food Sci. Hum. Wellness.

[25] Bang J.S., Jin Y.J., Choung S.-Y. (2020). Low molecular polypeptide from oyster hydrolysate recovers photoaging in SKH-1 hairless mice. Toxicol. Appl. Pharmacol..

[26] Park S.K., Van Hien P., Van Luong H., Yan S.-W., Byun S.Y. (2014). Peptide Hydrolysates from Astragalus membranaceus Bunge Inhibit the Expression of Matrix Metalloproteinases in Human Dermal Fibroblasts. KSBB J..

[27] Ye Y., You L., Deng Q., Li X., Zhao M. (2019). Preparation, structure identification and the anti-photoaging activity of peptide fraction OP-Ia from Ostrea rivularis. Rsc Adv..

[28] Mo Q., Li S., You S., Wang D., Zhang J., Li M., Wang C. (2022). Puerarin Reduces Oxidative Damage and Photoaging Caused by UVA Radiation in Human Fibroblasts by Regulating Nrf2 and MAPK Signaling Pathways. Nutrients.

[29] Ahmed T., Sun X.H., Udenigwe C.C. (2022). Role of structural properties of bioactive peptides in their stability during simulated gastrointestinal digestion: A systematic review. Trends Food Sci. Technol..

[30] Xu D., Wang W., Liao J., Liao L., Li C., Zhao M. (2020). Walnut protein hydrolysates, rich with peptide fragments of WSREEQEREE and ADIYTEEAGR ameliorate UV-induced photoaging through inhibition of the NF-kappa B/MMP-1 signaling pathway in female rats. Food Funct..

[31] Iosageanu A., Ilie D., Craciunescu O., Seciu-Grama A.-M., Oancea A., Zarnescu O., Moraru I., Oancea F. (2021). Effect of Fish Bone Bioactive Peptides on Oxidative, Inflammatory and Pigmentation Processes Triggered by UVB Irradiation in Skin Cells. Molecules.

[32] Chaoting W., Jixian Z., Haihui Z., Yuqing D., Haile M. (2020). Plant protein-derived antioxidant peptides: Isolation, identification, mechanism of action and application in food systems: A review. Trends Food Sci. Technol..

[33] Zhang C., Lv J., Qin X., Peng Z., Lin H. (2022). Novel Antioxidant Peptides from Crassostrea Hongkongensis Improve Photo-Oxidation in UV-Induced HaCaT Cells. Marine Drugs.

[34] Carvalho B.G., Raniero L.J., Martin A.A., Favero P.P. (2013). Phenylalanine ab initio models for the simulation of skin natural moisturizing factor. Spectrochim. Acta Part A Mol. Biomol. Spectrosc..

[35] Xiao J., Liu B., Zhuang Y. (2019). Effects of rambutan (*Nephelium lappaceum*) peel phenolics and Leu-Ser-Gly-Tyr-Gly-Pro on hairless mice skin photoaging induced by ultraviolet irradiation. Food Chem. Toxicol..

[36] Yathisha U.G., Bhat I., Karunasagar I., Mamatha B.S. (2019). Antihypertensive activity of fish protein hydrolysates and its peptides. Crit. Rev. Food Sci. Nutr..

[37] Mohankumar T., Chandramohan V., Lalithamba H.S., Jayaraj R.L., Kumaradhas P., Sivanandam M., Hunday G., Vijayakumar R., Balakrishnan R., Manimaran D. (2020). Design and molecular dynamic investigations of 7, 8-dihydroxyflavone derivatives as potential neuroprotective agents against alpha-synuclein. Sci. Rep..

[38] Connelly P.R., Snyder P.W., Zhang Y., McClain B., Quinn B.P., Johnston S., Medek A., Tanoury J., Griffith J., Walters W.P. (2015). The potency–insolubility conundrum in pharmaceuticals: Mechanism and solution for hepatitis C protease inhibitors. Biophys. Chem..

[39] de Almeida L.G.N., Thode H., Eslambolchi Y., Chopra S., Young D., Gill S., Devel L., Dufour A. (2022). Matrix Metalloproteinases: From Molecular Mechanisms to Physiology, Pathophysiology, and Pharmacology. Pharmacol. Rev..

[40] Liping S., Qiuming L., Jian F., Xiao L., Yongliang Z. (2018). Purification and Characterization of Peptides Inhibiting MMP-1 Activity with C Terminate of Gly-Leu from Simulated Gastrointestinal Digestion Hydrolysates of Tilapia (*Oreochromis niloticus*) Skin Gelatin. J. Agric. Food Chem..

[41] Lee K.E., Bharadwaj S., Yadava U., Kang S.G. (2020). Computational and In Vitro Investigation of (-)-Epicatechin and Proanthocyanidin B2 as Inhibitors of Human Matrix Metalloproteinase 1. Biomolecules.

[42] Xiao Z., Yang S., Liu Y., Zhou C., Hong P., Sun S., Qian Z.J. (2022). A novel glyceroglycolipid from brown algae Ishige okamurae improve photoaging and counteract inflammation in UVB-induced HaCaT cells. Chem. Biol. Interact..

[43] Selvaraj G., Kaliamurthi S., Thiruganasambandam R. (2016). Molecular docking studies of rutin on matrix metalloproteinase. Insights Biomed..

[44] Brodkorb A., Egger L., Alminger M., Alvito P., Assuncao R., Ballance S., Bohn T., Bourlieu-Lacanal C., Boutrou R., Carriere F. (2019). INFOGEST static in vitro simulation of gastrointestinal food digestion. Nat. Protoc..

